# Structure and compositional analysis of aluminum oxyhydroxide adsorbed pertussis vaccine

**DOI:** 10.1016/j.csbj.2020.12.023

**Published:** 2020-12-23

**Authors:** Jessica Duprez, Kristen Kalbfleisch, Sasmit Deshmukh, Jessie Payne, Manjit Haer, Wayne Williams, Ibrahim Durowoju, Marina Kirkitadze

**Affiliations:** a Analytical Sciences, Sanofi Pasteur Canada, 1755 Steeles Avenue West, Toronto, Ontario, Canada; b Department of Biology, York University, 4700 Keele Street, Toronto, Ontario, Canada; c Department of Physiology & Pharmacology, and Paediatrics, University of Calgary, 2500 University Drive NW, Calgary, Alberta, Canada; d SGS Canada, Biopharmaceutical Services, 6490 Vipond Drive, Mississauga, Ontario, Canada

**Keywords:** Pertussis, Tdap vaccines, TLR agonist, Particle sizing, FTIR, PAT

## Abstract

**Purpose:**

The goal of this study was to characterize an acellular pertussis vaccine (Tdap) containing genetically modified pertussis toxin (gdPT) and TLR agonist adsorbed to AlOOH adjuvant.

**Methods:**

Several analytical tools including nanoDSF, FTIR, and LD were used to examine the conformation of novel gdPT and the composition of AlOOH adjuvant formulations adsorbed to pertussis vaccine.

**Results:**

DLS particle size results were 9.3 nm and 320 nm for gdPT. For pertussis toxoid (PT), the DLS particle size results were larger at ~440 nm. After adsorption to AlOOH, which was driven by the protein antigen, the size distribution ranged from 3.5 to 22 µm. Two thermal transitions were observed by DSC for gdPT at 70 °C and 102 °C. The main thermal transition was confirmed to be at 72 °C by nanoDSF. All three vaccine formulations showed one thermal transition: Tdap-AlOOH had a thermal transition of 74.6 °C, Tdap-E6020-AlOOH had a thermal transition at 74.2 °C, and Tdap-CpG-AlOOH had a thermal transition at 77.0 °C. Analysis of pertussis toxin (PTx) and gdPT was also performed by FTIR spectroscopy for the purpose of comparison. The second derivative of the FTIR spectra showed an additional feature for PTx at 1685 cm^−1^ compared to gdPT. The antigen’s amide I and II regions were largely unchanged after adsorption to AlOOH adjuvant as shown by FTIR, suggesting that there were no significant changes in the secondary structure.

**Conclusion:**

gdPT conformation was successfully characterized using an array of analytical methods. All three Tdap formulations have similar thermal stability as shown by nanoDSF, similar size distribution as shown by LD, and similar overall secondary structure as shown by FTIR. In-line particle sizing and IR can be used as in-process characterization tools to monitor consistency of adsorbed vaccine and to confirm product identity.

## Graphical abstract

**Figure:**
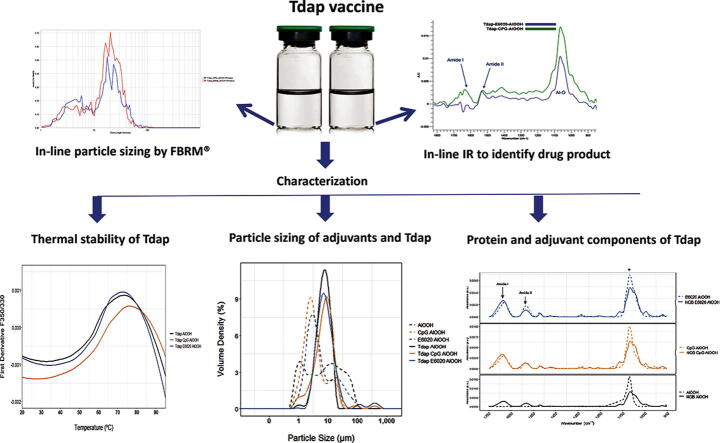

## 1 Introduction

Pertussis, or whooping cough, is an acute and highly contagious respiratory disease caused primarily by *Bordetella pertussis*. Prior to the implementation of immunization programs pertussis was highly endemic [1]. Vaccination was shown to be the most effective strategy to decrease the number of pertussis cases [2]. Sanofi Pasteur manufactures and distributes one whole-cell (wP) and two acellular pertussis (aP) vaccine families. Current aP vaccines (commonly named Tdap) are typically based on the following virulence factors: pertussis toxin (PT), filamentous hemagglutinin (FHA), pertactin (PRN), and fimbrial agglutinogen 2 and fimbrial agglutinogen 3 (FIM2/3 or FIM).

Adjuvants based on aluminum salts are frequently used in vaccines to boost immune response against infectious agents. Since most highly purified recombinant antigens are poorly immunogenic, adjuvants are often required to increase the level and duration of protection induced by vaccines [3]. Aluminum adjuvants induce weak Th1 and Th17 responses that may be necessary for the induction of protective immunity against certain diseases, such as pertussis [4]. Adsorption of immunostimulatory molecules to aluminum adjuvants limits the systemic distribution of the molecules which reduces the risk of systemic side-effects and enhances the targeting of such molecules and co-adsorbed antigens to antigen-presenting cells [5].

Immunostimulatory molecules, such as ligands for pattern recognition receptors, or more specifically, Toll-like receptors (TLRs), are excellent candidates for combination with adjuvants [6]. An example of a TLR ligand is a CpG oligonucleotide, which binds to TLR9. Cationic CpG is negatively charged and strongly adsorbs to aluminum oxyhydroxide (AlOOH) adjuvant. Another example of a TLR ligand is E6020, a modified natural lipid derived from enterobacterial lipopolysaccharides, which was found to be an agonist of TLR4. New combinations of adjuvants and antigens were formulated to enhance immunogenicity compared to the current Tdap vaccine. These new formulations include either the CpG or E6020 TLR agonists. After adding a TLR4 or TLR9 agonist to the Tdap vaccine, the immune response in mice induced a lower IL5 response [7], a hallmark of a Th2 oriented response [7]. It appears that this modulated T-helper cell profile is associated with accelerated *B. pertussis* clearance in mice [7]. In addition to the use of novel adjuvants and gdPT, other strategies are pursued to prevent nasal colonization of Bordetella pertussis by priming respiratory tissue-resident memory T cells that maintain long-term immunity at mucosal sites [8]. These include pertussis outer membrane vesicle, i.e., OMV vaccines for pertussis with nasal delivery systems that showed promising results in animal models and more limited early clinical trials [9]. The protection was associated with the induction od mucosal IL-17 and IFN-γ, increased lung and nasal IgA combined with strong systemic Th17 responses [9].

The Tdap vaccine formulation in this study contains the same antigens as the current aP formulation, with the exception of pertussis toxoid which was replaced with a genetically modified pertussis toxin (gdPT) [10], [11].

In this study, we characterized three formulations of Tdap vaccine in which the novel gdPT was used. The three formulations differ in TLR combinations: AlOOH adjuvant alone, AlOOH adjuvant with adsorbed TLR4 agonist (E6020) and AlOOH adjuvant with adsorbed TLR9 agonist (CpG). Based on previous experience with adsorbed vaccines, the array of analytical tools used in this study aims to examine product attributes at the purified protein stage [12], [13], as well as the adsorbed drug substance and drug product stages [14], [15], [16], [17], [18]. As discussed previously [19], characterization of vaccine attributes at both the drug substance and drug product stages have progressively higher criticality with respect to product supply, safety, and immunogenicity. For vaccines, this encompasses not only protein antigens, but also adjuvants, adsorbed antigens, and multivalent product formulations. Factors that can affect safety, efficacy, critical quality attributes, and critical material attributes may include, but are not limited to, protein adsorption and conformation and size distribution of adsorbed drug substances. The physio-chemical properties of adjuvants are important for the interaction between adjuvant and antigen. Adjuvants are prone to aggregation, which adversely affects their function and efficacy [20]. Therefore, investigating the morphology and defining the structure of formulated vaccines is crucial [20]. Our previous two studies were focused on H4-IC31 vaccine candidate, where IC31, an adjuvant consisting of peptide and oligonucleotide mixture, was used [17] and on the Quadracel™ vaccine, for which the aluminum phosphate adjuvant was used [21]. In the study presented here, AlOOH was used as the adjuvant for Tdap vaccine candidate formulations.

Similar to aluminum phosphate [21], AlOOH can alter protein conformation [15], [17], [18], [22] by either having a stabilizing [23], neutral [18], or destabilizing effect [14], [22], [24], [14]. This highlights the importance of analytical tools capable of monitoring changes in antigen conformation throughout the manufacturing process. The panel of methods used to examine the conformation of novel antigen gdPT included differential scanning calorimetry (DSC), differential scanning fluorescence (nanoDSF), dynamic light scattering (DLS), and Fourier transform infrared (FTIR) spectroscopy. These non-routine characterization tests were applied for the purpose of product knowledge. Since particle size can be an indication of both process consistency and product stability [24], [25], laser diffraction (LD) was utilized to characterize the size of adjuvant E6020-AlOOH, CpG-AlOOH, AlOOH, and adjuvanted drug product Tdap-E6020-AlOOH, Tdap-CpG-AlOOH, and Tdap-AlOOH. As protein conformation may affect the presentation of epitopes, the effect of adsorption on protein higher order structure was analyzed. FTIR was utilized to analyze secondary structure content and nanoDSF to examine higher order structure and thermal stability of adsorbed drug product. Although various multivalent vaccines may contain similar antigen profiles, minor variations in their composition or formulation may be detected by a sufficiently sensitive and selective method. FTIR used to derive signature spectra for the multivalent vaccines [21]; thus, it was employed for the analysis of AlOOH adsorbed Tdap vaccine.

Monitoring of vaccine formulations in-line throughout the manufacturing process is can be used for product knowledge and for acceleration of vaccine development. Process analytical technology (PAT) can be used for inline monitoring of material attributes, critical quality attributes, to enable real time characterization of vaccine formulations. It has been previously demonstrated that in-line PAT can be used to monitor particle size and chemical composition for the various stages of adjuvant manufacturing from raw materials through intermediate to final adjuvant product stage [26]. In this study, a feasibility of two in-line methods was assessed for their potential use in multivalent vaccine formulation. One of them is the in-line particle sizing method Focus Beam Reflectance Measurement (FBRM®), and another one is the in-line infrared ReactIR. Both in-line methods can be used to generate product trends and to model the process for better understanding and characterize the product in real time.

To summarize, the characterization of novel formulation of Tdap containing gdPT antigen and two TLR agonists, E6020 and CpG, adsorbed to AlOOH, is reported here for the first time. Two PAT solutions, in-line IR (ReactIR) and particle sizing (FBRM®) probes were investigated for characterization of vaccine formulation in-line.

## 2 Materials and methods

### 2.1 Reagents and materials

All samples used in this study were manufactured in-house with the exception of synthetic lipid E6020 acquired from Eisai Co. (Tokyo, Japan), and aluminum oxyhydroxide, AlOOH manufactured by Becton Dickinson (Mississauga, Canada). The Tdap vaccine formulations contained 4Lf/mL of Diphtheria Toxoid (DT), 10Lf/mL of Tetanus Toxoid (TT), 20 µg/mL of genetically modified Pertussis Toxin (gdPT), 10 µg/mL of Filamentous Haemagglutinin (FHA), 10 µg/mL of Pertactin (PRN) and 15 µg/mL of Fimbriae types 2 and 3 (FIM), and 0.66 mg/mL aluminum (AlOOH) with either 500 µg/mL of CpG ISS1018 (TLR9 agonist) or 10 µg/mL of E6020 (TL4).

The monovalent drug substances adsorbed onto AlOOH were prepared just for this study contained ~300 µg/mL of each, Diphtheria Toxoid and Tetanus Toxoid, whereas cP antigens were of ~100 µg/mL of each FIM, PRN, PTx, and gdPT.

### 2.2 Dynamic light scattering (DLS)

All DLS measurements of particle size distribution of pre-adsorbed gdPT antigen were performed using a Nanotrac 150 instrument (Microtrac, Montgomeryville, PA, USA). All samples were measured at room temperature at 20-fold dilution using MilliQ water, hence viscosity of water was used for the data analysis. Total volume for all measurements was 600 μL. Nanorange mode was enabled for appropriate analysis of the particle sizes below 20 nm. The data acquisition and analysis were done using Microtrac Flex software. The particle size was reported as hydrodynamic diameter in nm, to one decimal place. Coefficient of variation for the qualified generic DLS method ranged from 5 to 10% for gdPT.

### 2.3 Laser diffraction (LD)

All measurements of particle size distribution of adjuvant, adsorbed antigens and multivalent vaccine products were performed using a Mastersizer 3000 instrument (Malvern Instruments Ltd., Westborough, MA, USA), with an operating range of 0.01 to 3500.00 µm. Particle size distributions in solutions and suspensions were quantitatively determined by measuring the angular variation in intensity of light scattered from a laser beam passing through a dispersed particulate sample. The reportable value is Derived Diameter (Dv), which is the particle size (in μm) for a specific percentile of the cumulative size distribution. Particles were measured at room temperature without any prior sample preparation using the built-in “non-spherical” option within the software. All samples were tested neat, no preparation was required. Samples were added dropwise into the instrument until at least a 1.5% of obscuration was reached, and the average Dv10, Dv50 and Dv90 values of 5 measurements were reported in µm to one decimal point. The coefficient of variation for the qualified LD assay ranged from 5% to 7% for the adsorbed antigens.

### 2.4 Focused beam Reflectance measurement (FBRM®)

Focused beam reflectance measurement (FBRM®) is a real-time (in-line) monitoring tool for the determination of size and shape of the particles in the process by considering the chord length of the formed particles. FBRM® technique has a linear relationship with chord length distribution which is influenced by the geometry, size, number and dispersion of the particles. FBRM® technique can therefore be used for the determination of the particle size change kinetics in the fabrication of materials (e.g. adsorption reaction of protein antigens to AlOOH adjuvants, size of pre-adsorbed adjuvant, and drug product), thus providing an understanding of the material formation mechanisms.

Particle size data was determined using the ParticleTrack probe (Mettler Toledo Inc., USA) to exemplify a real-time measurement technique that can be further explored in-line to deepen process knowledge. This probe was submerged into the beaker containing a sample where particles in suspension could flow easily across the sapphire window. The samples were stirred continuously during the measurement using magnetic bar to maintain homogeneous dispersion of the suspended particles. Equipped with FBRM® technology, a laser beam was directed down a set of optics along the probe and was focused to a tight beam spot at the window. The rotating optics focused the beam, which then rapidly scanned across particles as they flowed past the window. The resulting light scattering pattern from the particles was detected by the probe and used to calculate the chord length, or distance across each particle. The reportable value in FBRM® is the chord length at percentile C, which is C50 (in µm) in this case. The real-time chord length distribution was monitored using iC FBRM® ParticleTrack software (Mettler Toledo Inc., USA).

### 2.5 Fourier transform infrared (FTIR) spectroscopy

FTIR spectroscopy was performed using a Vertex 70 FTIR Spectrometer (Bruker Optics, Bremen, Germany), equipped with a cryogenically-cooled MCT (mercury-cadmium-telluride) detector and a BioATRII sampling accessory. A sample volume of 20 µL was loaded onto the sample cell and the spectra were collected at a resolution of 0.4 cm^−1^ at 25 °C with a wavenumber accuracy of 0.01 cm^−1^ at 2000 cm^−1^. The samples were stabilized for 1 min on the ATR crystal. Background (Milli-Q water) and sample measurements were conducted with each reported measurement representing an average of 200 scans. Data acquisition and analysis were performed using the OPUS 6.5 software (Bruker Optics, Bremen, Germany). OPUS automatically subtracts the background signal from the sample to produce the spectrum for the analyte. All measurements were carried out at 25 °C using a Haake DC30/K20 temperature controller (Karlsruhe, Germany). After acquiring the FTIR spectra, the baseline was corrected by removing the scattering signal using the OPUS software. Quant2 software (Bruker Optics) was used to estimate secondary structure with an error of 5.5% for alpha-helix content and 4.4% for beta-sheet content. The second derivative spectrum was generated using the Savitzky-Golay algorithm, which allowed simultaneous smoothing of the spectrum. The purpose of second derivative was to examine subtle differences between the gdPT and PTx, and to detect beta-turns, which are not evaluated by Quant2 software for secondary structure estimation. Re-plotting were performed using SigmaPlot.

### 2.6 In-line FTIR probe

IR spectra were recorded using the ReactIR 702L (Mettler Toledo Inc., USA). ReactIR is a probe that permits the visualization of the adsorption reaction progression over time, providing highly specific information about initiation, endpoint, conversion, kinetics, secondary structure changes, mechanism, and pathway. The real-time, in situ, mid-infrared system, ReactIR system directly follows the concentration of key reaction species as they change throughout the reaction and serves as an example of an in-line technique that can be further explored. This probe is equipped with an Attenuated Total Reflectance (ATR) sensor that measures the changes of the IR beam as it is internally reflected upon contact with the sample. The resulting beam was attenuated in the regions of the IR spectrum where the sample absorbed energy. This attenuated beam returned to the ATR crystal and exited the opposite end to be directed to the detector. The probe was inserted directly into the sample vessel where particles in suspension could flow easily across the diamond crystal. The software iCIR (Mettler Toledo Inc., USA) was programmed to collect single IR spectra for the various samples.

### 2.7 nanoDSF

The nanoDSF method was performed on a Prometheus NT.48 system (Nano Temper Technologies, Munich, Germany). nanoDSF uses intrinsic fluorescence, which is a dye-free method to evaluate changes in aromatic residues (fluorophores) within proteins in response to the changes in their local environment. The shift and intensity change in fluorescence emission is monitored, with a change in the intrinsic fluorescence indicating that the protein has unfolded. Thermal stability of protein is characterized using the melting temperature (T_m_), which indicates the point at which half the protein is unfolded. In the nanoDSF method, this is determined by using the ratio of fluorescence recorded at 330 nm and 350 nm; this ratio has shown to be more sensitive in detecting T_m_ as compared to the use of a single wavelength. Samples were filled in capillary tubes without any further preparation and excited at 285 nm with 20% power output. The thermal profiles were recorded from 20 to 95⁰ C with 2⁰ C/min scan rate.

## 3 Results

### 3.1 Particle sizing

**Fig. 1:**
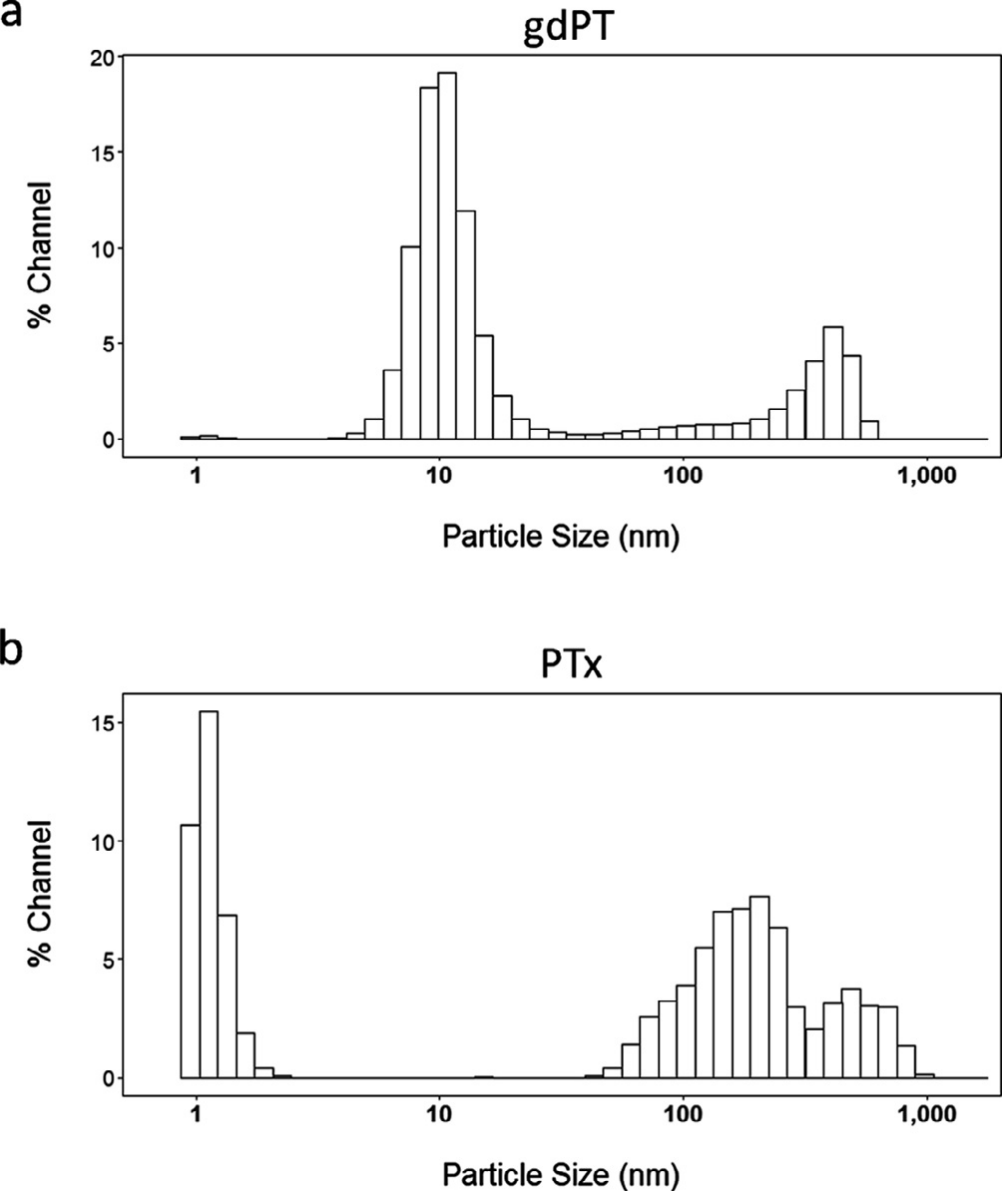
**a)** Particle size distribution of gdPT protein antigen as measured by DLS. **b)** Particle size distribution of PTx protein antigen as measured by DLS.

**Fig. 2:**
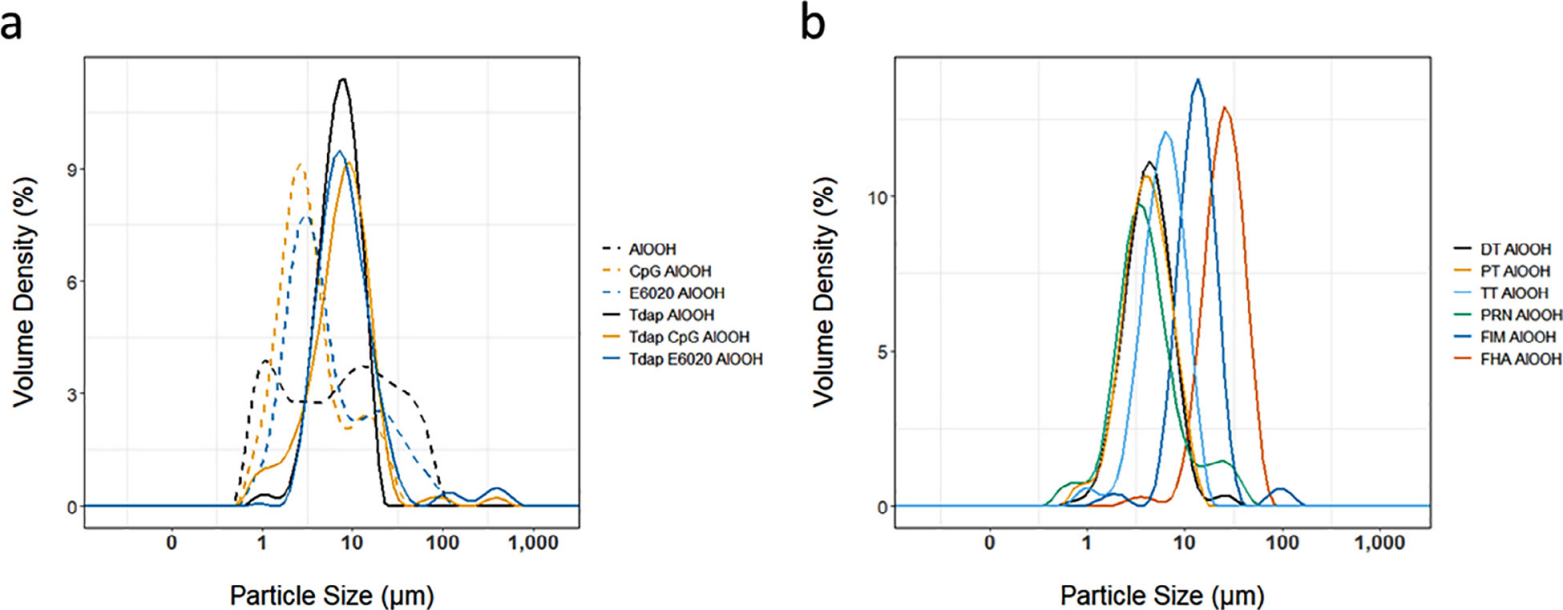
**a)** LD particle size distribution of adjuvants and Tdap adsorbed vaccine formulations. AlOOH (black dashed trace), E6020-AlOOH (orange dashed trace), CpG-AlOOH (blue dashed trace), Tdap-E6020-AlOOH (green trace), Tdap-CpG-AlOOH (blue trace) and Tdap-AlOOH (black trace). **b)** Particle size distribution of adsorbed protein antigens used in a previous formulation of Tdap vaccine and shown for comparison purposes: Diphtheria Toxoid (DT) (dark purple trace), Pertussis Toxoid (PT) (orange trace), Tetanus Toxoid (TT) (light blue trace), Pertactin (PRN) (green trace), Fimbriae (FIM) (blue trace), and Filamentous Haemagglutinin (FHA) (red trace). The size distribution of all adsorbed protein antigens is representative of one lot, five repeats. (For interpretation of the references to colour in this figure legend, the reader is referred to the web version of this article.)

Size distribution profiles of pre-adsorbed gdPT (Fig. 1), and adsorbed Tdap formulations were measured using LD (Fig. 2). The size distribution profiles as determined by DLS for each of the pre-adsorbed antigens were reported by Kalbfleisch et al [19]. These antigens will ultimately be formulated into a multivalent Tdap vaccine with protection against Pertussis, Diphtheria and Tetanus. On an average, monomeric gdPT ranged from 9.06 to 9.36 nm, whereas oligomers were detected in the range of 100–1000 nm. In contrast, PTx showed predominantly multimeric species.

Size distribution profiles of the adjuvant and vaccine formulations were measured using LD (Fig. 2). Both E6020-AlOOH and CpG-AlOOH had a similar particle size (Dv50) of 4.05 µm and 2.98 µm, respectively (Table 1). The multivalent vaccine formulation in AlOOH adjuvant had a particle size of 7.27 µm, and the particle sizes did not differ when formulated with either CpG or E6020 adjuvant were of ~8 µm in both cases (Table 1). As for the TLR agonists, the adsorption of proteins: DT, TT, PT, FIM, PRN, and FHA resulted in a different size distribution observed for all drug substances (Fig. 2a) and also different from AlOOH (Table 1).

**Table 1:** Particle size distribution of adsorbed protein antigens, adjuvants and Tdap vaccine formulations.

	**Dv10**	**Dv50**	**Dv90**
DT-AlOOH	3.26	5.71	9.62
TT-AlOOH	3.07	6.14	11.2
PT-AlOOH	2.51	4.53	9.44
PRN-AlOOH	2.13	4.08	10.7
FHA-AlOOH	12.4	23.2	43.5
FIM-AlOOH	7.44	12.1	19.3
AlOOH	0.769	3.99	28.3
CpG-AlOOH	1.31	2.98	14.0
E6020-AlOOH	1.65	4.05	28.6
Tdap-AlOOH	3.55	7.27	135
Tdap-E6020-AlOOH	3.51	8.03	21.9
Tdap-CpG-AlOOH	2.45	7.82	17.3

### 3.2 Secondary structure conformation and adjuvant

**Fig. 3:**
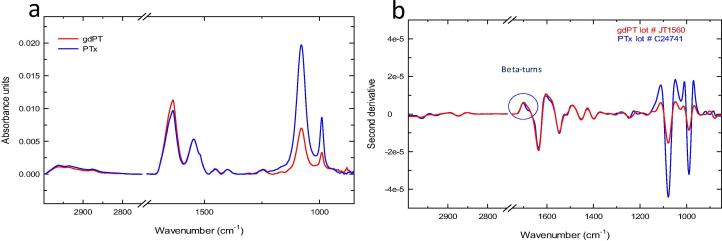
Overlay of FTIR spectra for gdPT (red trace) and PTx (blue trace). Calculated second derivative of FTIR spectra of gdPT (red trace) and Pertussis Toxin (PTx) (blue trace). (For interpretation of the references to colour in this figure legend, the reader is referred to the web version of this article.)

The FTIR spectroscopy was conducted on gdPT and PTx as shown in Fig. 3, with major peaks summarized in Table 2. Both samples showed similar spectral features, with the following band detected for: Amide I at 1636 cm^−1^, Amide II at 1546 cm^−1^, methyl deformation at 1453 cm^−1^ and 1400 cm^−1^, and phosphate contributions at 1078 cm^−1^ and 990 cm^−1^ (Fig. 3a).

**Table 2:** Major identifiable peaks.

**Sample**	**Major identifiable peaks (cm^−1^)**							
gdPT	1636	1546	1453	1400			1078	990
gdPT-AlOOH	1636	1546	1453	1400			1078	990
E6020-AlOOH	1630	1533	1470	1429	1287	–	1067	–
Tdap-E6020-AlOOH	1634	1533	1469	1427	1288	1112	1066	994
CpG-AlOOH	1632	1532	1470	1423	1289	–	1068	–
Tdap-CpG-AlOOH	1641	1535	1469	1422	1282	1112	1065	996
AlOOH	–	–	–	–	–	–	1068	–
Tdap-AlOOH	1633	1532	1469	1423	1290	1109	1065	996

FTIR and the Amide I band is widely used to quantify conformational changes in proteins, however there can be overlapping bands within the same regions which can be separated by calculating the second derivative of the spectra. By calculating the second derivative spectra, it was shown that PTx has an additional feature at 1685 cm^−1^ (circled in Fig. 3b) representing beta-turns for PTx. These two samples were compared to gain additional information only. Since these samples had different concentrations, the signal intensities were not compared, however the peak positions, additional spectral features or missing spectral features were scrutinized. In conclusion, the FTIR spectrum of gdPT yields additional information besides protein signature peaks, such as contributions from buffer matrix or any other component from the sample and its potential influence on the molecular structure. Adsorption to AlOOH showed no significant changes in Amide regions of gdPT, PRN, and FIM proteins. Whereas, a significant decrease of Amide II peak was seen for DT and TT, and a decrease of Amide I peak, and disappearance of Amide II peak was noted FHA. No changes were noted for PT, except the decrease of glycerol peak due to dilution by AlOOH.

**Fig. 4:**
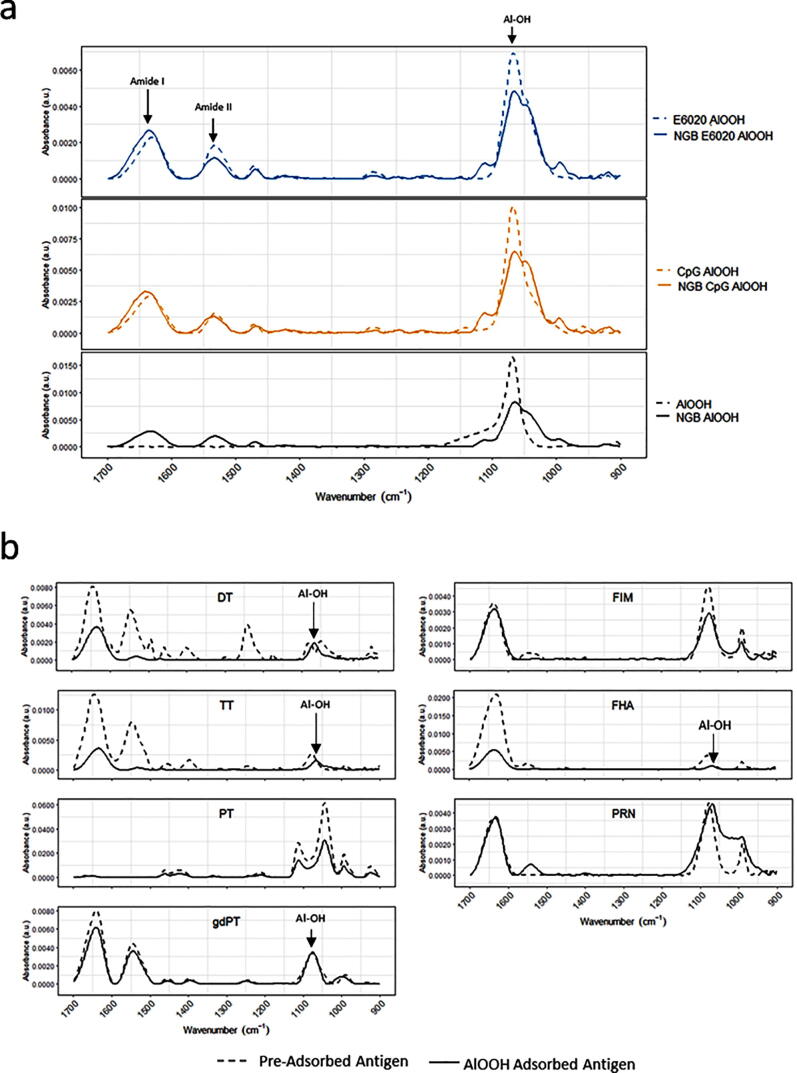
**a)** FTIR spectra of representative samples highlighting prominent absorptions bands from the different AlOOH and Tdap formulations, from 1700 cm^−1^ to 900 cm^−1^. a1) shows E6020-AlOOH adjuvant (dashed line) and Tdap-E6020-AlOOH formulations; a2) shows CpG-AlOOH adjuvant (dashed line) and Tdap-CpG-AlOOH formulations; a3) shows AlOOH alone (dashed) and Tdap AlOOH controls. Linear baseline correction with 42 iterations was applied to each spectrum to remove baseline drift. All formulations have an Al-O–H bending absorption band (1065 – 1068 cm^−1^), and all adsorbed samples have peaks in the Amide I (1600–1700 cm^−1^) and Amide II (1500–1600 cm^−1^) region. **b)** FTIR spectra of representative samples of pre-adsorbed antigens and their AlOOH absorbed counterparts, from 1700 cm^−1^ to 900 cm^−1^.

Three adjuvant formulations: AlOOH control, CpG-AlOOH, E6020-AlOOH were examined by FTIR (Fig. 4). When these are analyzed with a pure AlOOH control, adsorbed samples have peaks present in the amide I (1700–1600 cm^−1^) and amide II (1600–1500 cm^−1^) regions. However, these peaks are not affected by the absorption of either CpG or E6020, or of the Tdap multivalent vaccine antigens. AlOOH absorbs strongly in the Al-O–H stretch region (1065 cm^−1^), and although all AlOOH formulations have a band present at ~1065 cm^−1^, a difference can be seen within these peaks. Samples containing AlOOH without the multivalent vaccine have narrow peaks, whereas when Tdap is present the Al-OH band is broader with two shoulders/small peaks at 994–966 cm^−1^ and 1109–1112 cm^−1^ (Table 2).

All Tdap formulations contain the same antigens and AlOOH as an adjuvant. As a result, the spectral features of these combination products are quite similar (Fig. 4), yet detectable minute differences were observed. For instance, the peak representative of the P-O stretch (around 1065 cm^−1^) had higher absorbance in Tdap-CpG-AlOOH when compared to Tdap-E6020-AlOOH, and Tdap-AlOOH the latter showing a shift in the shoulder peak of 1112 cm^−1^ to 1109 cm^−1^.

### 3.3 Thermal stability

**Fig. 5:**
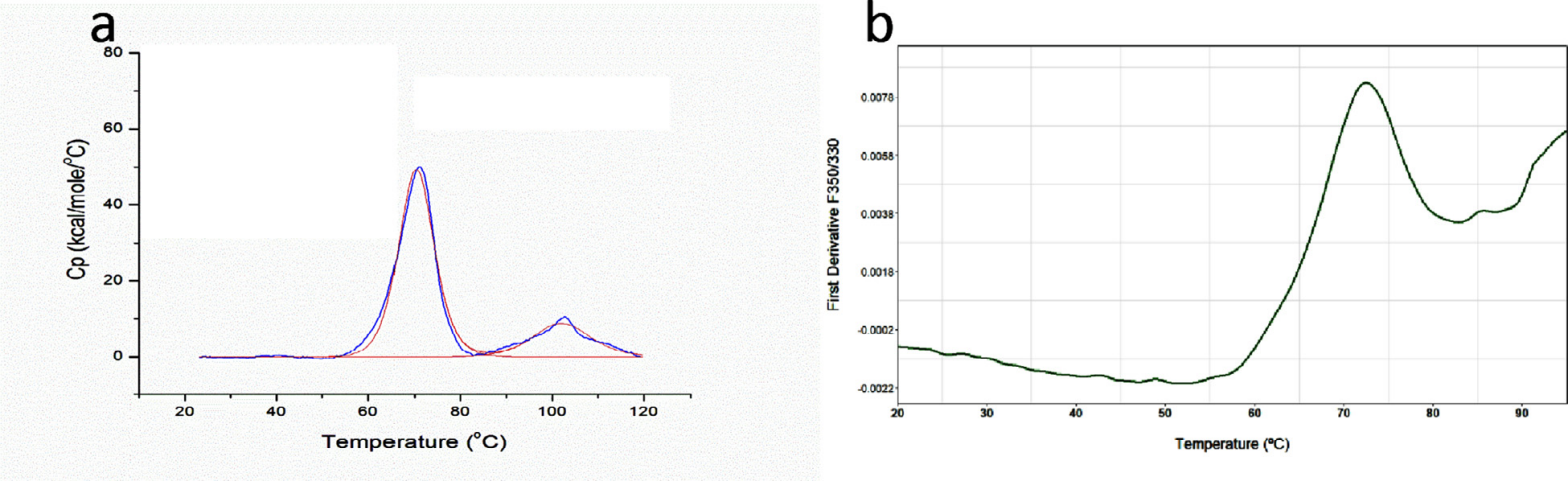
**a)** DSC thermogram of gdPT – experimental profile (blue trace) and fitted line (red line). Thermal transitions Tm_1_ of gdPT is 70.4 °C, and Tm_2_ is 102.1 °C. **b)** nanoDSF thermal profiles of gdPT (green trace), showing the first derivative of intrinsic fluorescence emission ratio (350 nm/330 nm). Thermal transition (Tm) of gdPT is 72.3 °C. (For interpretation of the references to colour in this figure legend, the reader is referred to the web version of this article.)

DSC was used as an orthogonal method to characterize the tertiary structure of the gdPT (Fig. 5a). Transition midpoints (T_m_) were collected for a total of 7 runs and the average transition midpoint temperatures T_m1_ and T_m2_ were 70.4 °C and 101.3 °C, respectively.

The main thermal transition of gdPT was confirmed by nanoDSF and observed at 72 °C (Fig. 5b). Intrinsic fluorescence emission ratio 350/330 nm increased after 90 °C, indicative of the second thermal transition also detected by DSC, however it is only partially captured due to the upper temperature limitations of the technique. In contrast to gdPT, PTx showed two peaks with lower Tm as previously reported by Krell et al [27].

**Fig. 6:**
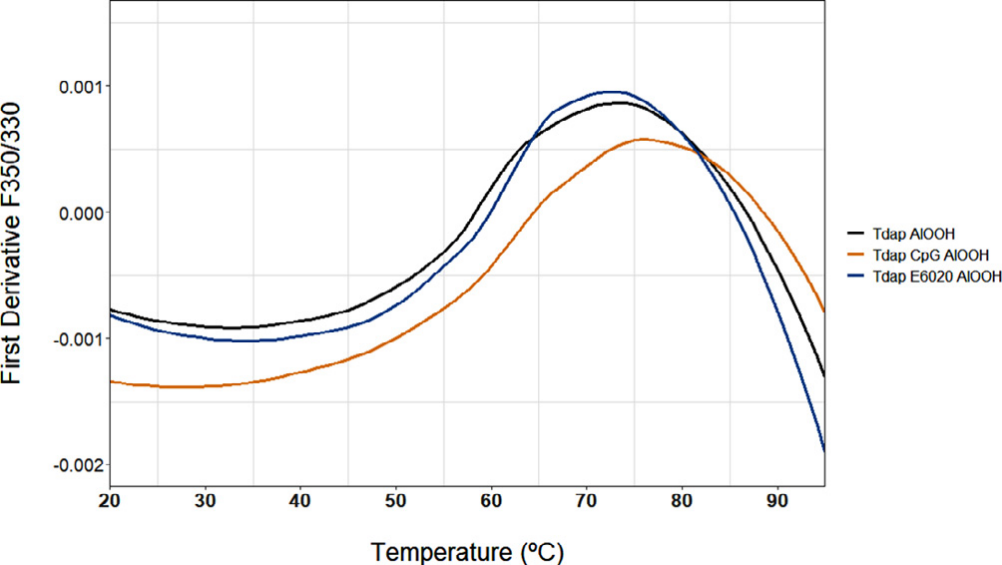
Thermal profiles of Tdap-AlOOH (black trace), Tdap-E6020-AlOOH (orange trace) and Tdap-CpG-AlOOH (blue trace), showing the first derivative of intrinsic fluorescence emission ratio (350 nm/330 nm). (For interpretation of the references to colour in this figure legend, the reader is referred to the web version of this article.)

The tertiary structure and thermal stability of the different vaccine formulations can be examined by nanoDSF, to assess if any difference conformation can be detected between different adjuvant formulations. For all vaccine formulation one thermal transition was detected (Fig. 6). The Tm values are summarized in Table 3. Tdap-E6020-AlOOH formulation showed similar Tm to that of Tdap-AlOOH, whereas Tdap-CpG-AlOOH showed Tm of 3 °C greater compared to the other two. Considering that nanoDSF qualification showed %CV of only 0.5 °C, this difference was statistically significant.

**Table 3:** Thermal transition of gdPT and Tdap vaccine formulations by nanoDSF.

Sample		Average Temperature (°C)
gdPT	T_m_	72.3
Tdap-AlOOH	T_m_	74.6
Tdap-CpG-AlOOH	T_m_	77.0
Tdap-E6020-AlOOH	T_m_	74.2

The CpG containing Tdap formulation had a minimally elevated transition temperature when compared to the other formulations. These data shows a consolidated thermal unfolding of four proteins components, gdPT (72 °C), Diphtheria Toxoid (80 °C), Tetanus Toxoid (78 °C), and Pertactin (68 °C and 80 °C) with an average Tm of 74 °C or 77 °C. The other proteins, Filamentous Haemagglutinin (FHA) and Fimbriae (FIM) do not contribute to the signal. Chemically modified FHA used for the formulation does not unfold in the range of 20 °C − 100 °C, where FIM is a fibrillar protein that does not contain a hydrophobic core to unfold.

## 4 Discussion

The formulation of combinatory vaccines contains multiple components inlcuding proteins, adjuvants, and excipients, all of which can form complex interations within the matrix. Characterization of individual components in conjuction with their complexes is imperative for process and product knowledge and can be drawn on for quality control. This characterization includes, but is not limited to, compositional and structural analysis and identity. As per ICH Q6B, it is important to understand and characterize physio-chemical properties of protein antigens including higher order structure, purity, identity, biological activity, and post-translational modifications [28]. This study focuses on the higher order structure of vaccine components and determining vaccine identity by biophysical methods.

Particle size has implications on the uptake of particles by antigen presenting cells [25]. The particle size of the three Tdap vaccine formulations are a similar size of approximately 8 µm. This is close to the optimal particle size for the successful uptake by antigen presenting cells [25]. The size distribution of AlOOH observed in this study agreed with previously reported data [6]. It has been shown previously that AlOOH nanoparticles are elongated and form loosely connected porous aggregates that vary in size from 1 to 20 µm [6]. This study showed that adsorption of proteins and TLR agonists causes a rearrangement of AlOOH particles, resulting in a different size distribution observed for all drug substances (Fig. 2b) and CpG and E6020 containing adjuvants (Fig. 2a). The results also indicated that particle size of all drug substances was greater compared to AlOOH. Therefore, it appears that the size of the adjuvants was driven by the TLD agonists adsorbed onto AlOOH, whereas the size of drug substances were defined by the protein antigens adsorbed to AlOOH. In contrast, our previous study showed that particle size of the vaccine components DT, TT, PT, FIM, PRN, and FHA adsorbed to aluminum phosphate is defined by size of aluminum phosphate itself [18]. It is therefore important to characterize particle size of antigens pre and post adsorption to AlOOH to ensure lot-to-lot consistency. Although the formulation and immunological response of adjuvants E6020-AlOOH [29], [30] and CpG-AlOOH [31] have been previously studied, this is the first time the adjuvant containing TLR agonists adsorbed to AlOOH were characterized physio-chemically with techniques that can be directly applied in the manufacturing unit of operation to test particle size and composition of adsorbed drug substances and drug product in-line.

FTIR spectroscopy was used to examine changes of the Al-O–H band and secondary structure in protein antigens as a result of adsorption (Fig. 4b). Pre-adsorbed antigens were compared to their AlOOH absorbed formulation (Fig. 4b). Within the FTIR spectra, individual peaks represent vibrational modes of the molecules and the alteration in the local environment of these molecules is detected by peak change. Secondary structure elements of DT, TT, FHA, PRN, PT, and FIM detected by FTIR (Fig. 4b) were consistent with the structure of PRN [32], Diphtheria Toxin [33], Tetanus Toxin [34], Pertussis Toxin [35], and with the models of FHA [36], [37] and FIM [37] reported in literature. The FTIR spectra of monovalent DT, TT, PT, FHA, PRN, and FIM drug substances consisting of single antigens were compared with the spectra of the Tdap vaccine formulation. In the process used to formulate the Tdap vaccine, no monovalent drug substances were produced, as all antigens were adsorbed in conjunction with one another. Since gdPT was used in Tdap formulations, this comparison is relevant for the DT, TT, FHA, PRN, and FIM drug substances adsorbed to AlOOH. In addition, the gdPT antigen showed variation in the FTIR spectrum (Fig. 3) compared to a pre-adsorbed PT spectrum previously captured [21]. Since the latter is chemically modified, its structure and thermal stability is different from gdPT and therefore cannot be directly compared. Hence, a comparison between gdPT and pertussis toxin, PTx, was performed (Fig. 3) and revealed similar spectral features. However, using the second derivative of the spectra detected an additional feature at 1685 cm^−1^ for PTx. Circular Dichroism (CD) was used as an orthogonal method to verify these results and showed that gdPT and PTx spectra had minima at 208 nm (Fig. S1); however, there was no pronounced minimum at 222 nm. This indicates an altered secondary structure pertaining to α-helices. On the other hand, the PTx sample showed presence of α-helical content. The CD spectra for all three samples were recorded to gain comparative knowledge of secondary structure of the protein. The results are consistent with the recently reported crystal structure for gdPT [38] and showed that it is nearly identical to that of PTx. Although gdPT showed presence of monomer and multimer in solution, where PTx had only multimeric species, the X-ray analysis [35] demonstrated that they both consist of five subunits, referred to as S1, S2, S3, S4, and S5 [38]. In addition, hydrogen–deuterium exchange mass spectrometry revealed distal changes in the S2-S5 subunit interactions resulting in tighter packing of B-oligomer and leading to increased thermal stability [38]. The latter is consistent with Tm of ~70 °C as observed by DSC (Fig. 5a) and nanoDSF analysis (Fig. 5b). In addition, gdPT protein has a monomer and oligomer as reported by DLS (Fig. 1a), whereas PTx (Fig. 1b) and PT have oligomer only [19]. The thermal stabilities of Tdap vaccine formulations were similar (Fig. 6). Tdap-AlOOH and Tdap-E6020-AlOOH showed the same Tm of 74 °C, whereas Tdap-CpG-AlOOH showed slightly higher Tm of 77 °C (Fig. 6). This is likely due to the stabilizing effect of CpG (Fig. 6).

FTIR spectra of Tdap adsorbed AlOOH formulations (Fig. 4) contain rich information that can be used for in-process testing to verify vaccine bulk drug product identity prior to filling. These observations were similar to those observed for AlPO_4_ adsorbed vaccines reported previously [21], [26], [29].

Particle sizing for determining the size of adjuvant and adjuvanted drug product and secondary structure characterization using FTIR can be further studied in-line during the adsorption process with PAT. This allows for monitoring the progress of the reaction and facilitates a better understanding of the product. In-line FTIR technology is sufficiently sensitive to observe secondary structure of adjuvanted drug product (Fig. S2). Similar to the off-line FTIR results previously discussed, the peaks from the Al-OH bond, amide I region, and amide II region were all visible (Fig. S2). Furthermore, differences were observed between Tdap-E6020-AlOOH and Tdap-CpG-AlOOH (Fig. S2). This suggests that the FTIR probe is a method that can distinguish between these two formulations. Tdap-E6020-AlOOH presented a lower magnitude of absorbance units at the Al-OH bond stretch and in the amide I region compared to Tdap-CPG-AlOOH (Fig. S2). This is likely due to the conformation differences between the two samples. Tdap-CPG-AlOOH had a higher magnitude of absorbance units in this region (Fig. S2). The off-line FTIR spectrometer previously discussed showed similar wavenumbers (cm^−1^) for the Al-OH, Amide I, and Amide II peaks, providing orthogonal verification for this measurement. Likewise, the FBRM® results showed that minor differences between Tdap-CPG-AlOOH and Tdap-E6020-AlOOH can be detected (Fig. S3). These results are comparable to those observed with LD, with similar bimodal shape observed in the particle size distributions (Fig. S3). In general, these results suggest that in-line ReactIR and FBRM® can be applied to these processes to observe changes occurring in the particle size and secondary structure in real-time, facilitating lot-to-lot consistency and furthering process understanding.

## 5 Conclusions

In this study, conformation of genetically detoxified Pertussis Toxin, gdPT, was examined by a panel of analytical techniques for product knowledge. gdPT exists predominantly in a monomeric form with a hydrodynamic radius of 9.3 nm, with some residual oligomeric content. gdPT is thermally stable and has a main denaturation transition at ~70 °C to72°C as detected by DSC and nanoDSF. Further analysis by FTIR and CD demonstrated secondary structure content consistent with a mix of alpha helical and beta sheet structure, which is comparable to wild type PTx.

Novel antigen gdPT was used in all three formulations of Tdap vaccine examined in this study. This provided downstream comparison of overall characterization as a result of process and formulations. Three formulations of Tdap vaccine, Tdap-AlOOH, Tdap-E6020-AlOOH, and Tdap-CpG-AlOOH, showed similar particle size, thermal stability, and overall secondary structure as shown by LD, nanoDSF and FTIR, respectively. Excitingly, the latter can be used as a lean technique to confirm product identity.

## Declaration of Competing Interest

The authors declare that they have no known competing financial interests or personal relationships that could have appeared to influence the work reported in this paper.
