# Supplementary material for: Structure and compositional analysis of aluminum oxyhydroxide adsorbed pertussis vaccine
Source: Comput Struct Biotechnol J. 2020 Dec 23;19:439–47. doi: 10.1016/j.csbj.2020.12.023 (PMC7804342; doi:10.1016/j.csbj.2020.12.023)
Supplement: Supplementary data 1 [file mmc1.docx]

## Supplement for Tdap – AlOOH adsorbed vaccine manuscript

**Figure S1.** The far-UV CD spectra overlay of gdPT, PTx and PT samples


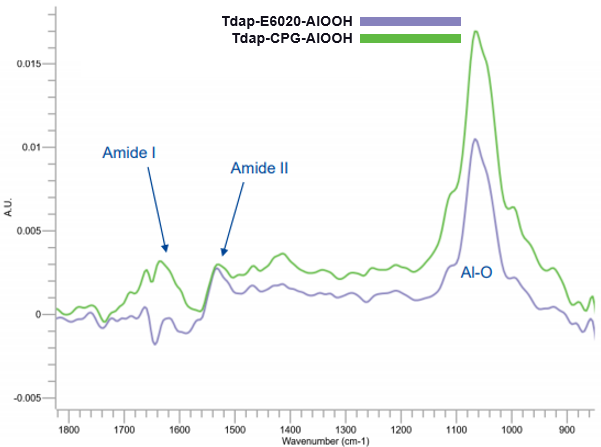


**Figure S2: ReactIR Probe Analysis of Tdap-E6020-AlOOH and Tdap-CPG-AlOOH**


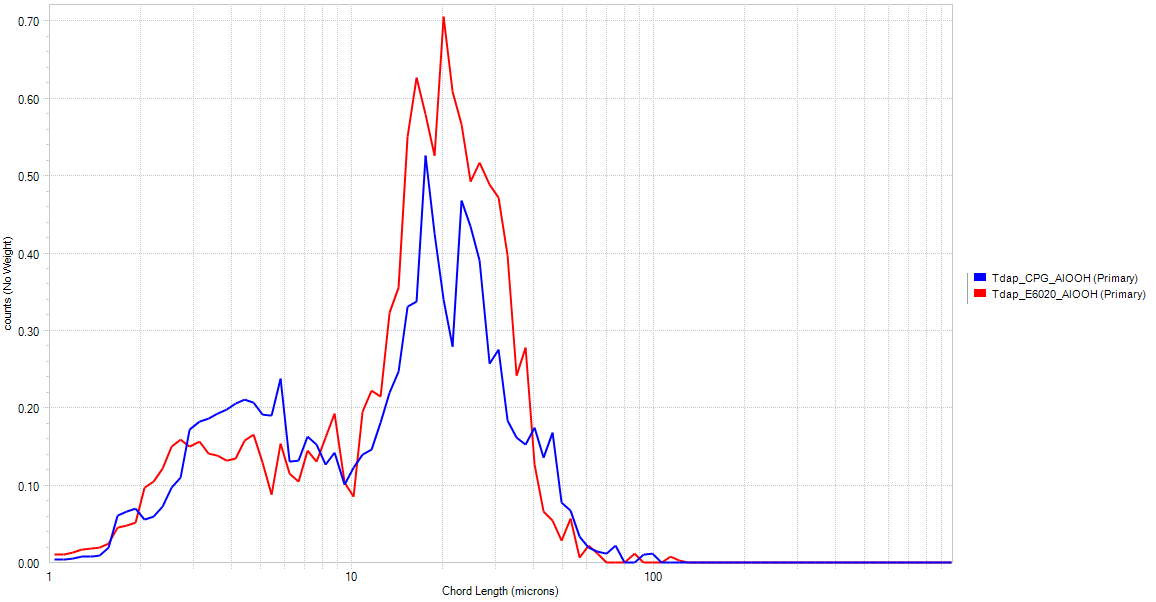


**Figure S3: FBRM^®^ Probe Analysis of Tdap-E6020-AlOOH and Tdap-CPG-AlOOH**
